# The Effect of Wearing a Customized Mouthguard on Body Alignment and Balance Performance in Professional Basketball Players

**DOI:** 10.3390/ijerph17176431

**Published:** 2020-09-03

**Authors:** Hae Joo Nam, Joon-Hee Lee, Dae-Seok Hong, Hyun Chul Jung

**Affiliations:** 1 Department of Health Rehabilitation, O-san University, 45 Cheonghak-ro, Osan-si, Gyeonggi-do 18119, Korea; tjlove@osan.ac.kr (H.J.N.); spoho@osan.ac.kr (D.-S.H.); 2 Department of Coaching, College of Physical Education, Kyung Hee University (Global Campus), 1732 Deokyoungdaero, Giheung-gu, Yongin-si, Gyeonggi-do 17014, Korea; jhc@khu.ac.kr

**Keywords:** body alignment, static balance, dynamic balance, basketball, mouthguard

## Abstract

The present study examined the influence of a customized mouthguard on body alignment and balance performance in professional basketball players. Twenty-three professional male basketball players, aged 25.8 ± 8.6 years old, were voluntarily assigned to participate in three treatments, including no treatment (no mouthguard), acute treatment (wearing a mouthguard), and repeated treatments (8 weeks follow-up). Body alignment status, such as spinal and pelvic posture and balance performance, were measured at each time point using a 3D Formetric III (Germany) and a postural control device (Posturomed 202, Germany), respectively. A repeated MANOVA analysis with a Bonferroni post hoc test was applied, and the adjusted *p*-value was set at 0.02. No significant treatment effect was observed in body alignment (*p* = 0.302). However, univariate analysis showed a significant difference in pelvic torsion, where it was decreased after acute and repeated mouthguard treatments compared to no treatment (*p* < 0.001). Kyphotic angle also increased significantly following 8 weeks of treatment compared to no treatment (*p* < 0.001) and acute treatment (*p* < 0.002). There was a significant treatment effect on balance performance (*p* < 0.001). Both static and dynamic balance performance improved following 8 weeks of treatment (*p* < 0.001). Our study revealed that a customized mouthguard provides a benefit to balance performance. Notably, repeated treatment impacts on balance performance more than acute treatment. Although our findings did not show a significant effect on body alignment, some positive results, such as pelvic torsion and kyphotic angle, may provide substantial information for developing future longitudinal studies with large sample sizes.

## 1. Introduction

Sports athletes commonly undergo highly intensive training to gain specialized physical strength and develop skills and specific movement patterns needed for competitions. However, unilateral training or incorrect movements can induce the muscular imbalance between the left and right sides of the body, which may interfere with well-balanced bodily development [1]. It is also known that asymmetric loads during repeated training can increase the risk of spinal deformation and structural scoliosis due to the unilateral mechanical action of force [2]. It has been reported that the prevalence of sports-related spinal deformity, such as functional scoliosis, was 33.5% in a sample of 571 athletes [3]. Such spinal deformity can alter the characteristics of spinal muscles, including muscle spindle activity [4], which interferes with the interactions between various body components [5]. Consequently, this causes instability when maintaining a standing posture, while also affecting balance performance [6].

Muscular imbalance throughout the asymmetrical movements negatively influence body alignment [7]. In particular, the misaligned spinal column can cause back pain and negatively impact on athletic performance in professional athletes [8,9]. For instance, basketball training involves numerous movements, such as rapid acceleration and deceleration, as well as continued jumping movements. Athletes also require higher physiological demand (77–95% HRmax) with intermittent movements to rapidly transit from defense to offense motions [10]. They often experience muscular injuries, including ankle and lumber sprains, when eccentric load is applied on the legs during pivoting and jumping. Sudden deceleration while changing direction, bad landing after jumping, and failed direction control have been also reported as major causes of injury [11]. The athlete’s lumbar pain is mainly characterized by “postural back pain”, which leads to an asymmetrical position by repeating excessive training for a long period of time, and the deformed body alignment causes dislocation when the load is concentrated in the local region of the spine. Thus, it makes it difficult to maintain normal spinal curvature. The deformed body alignment can affect the core muscles’ strength and a decrease in the range of motion reduces the extensor strength [12]. In addition, when the hip joint is displaced, there is a difference in the height of the left and right sides of the pelvis, and the lengths of both legs are changed; thus, the balance of the pelvis is collapsed and makes it difficult to maintain the midline of the spine [13].

To safely protect physical functions from undesirable movement patterns, it is important to maintain proper positional state and posture in each part of the body. In particular, proper position and posture of the head and neck may play an important role in postural maintenance and function, where muscles in the head and neck area form a balance with each other to help perform movements by maintaining the posture of the spine connected to the area and controlling the mandibular movement. A previous case study reported that a female professional basketball player who suffered the temporomandibular joint dysfunction (TMJ) improved her postural control after six months of wearing an occlusal splint during training and competitions [14]. In the same way, Mannheimer and Rosenthal [15] reported that the position of the cervical spine changes temporomandibular joint (TMJ) disorder and changes the direction of the head and consequently changes the position of the mandible. Therefore, the position of the appropriate mandibular joint can improve the exercise ability when the movement occurs, and the muscle strength can be changed according to the position of the jaw [16]. Recently, there have been reports of improvements in performance by changing the position of the jaw joints after wearing a mouthguard [17,18,19]. However, a lack of studies may limit our understanding of the effectiveness of wearing a mouthguard on body alignment and balance performance in professional athletes. Therefore, the present study examined the acute and repeated effects of wearing a customized mouthguard on body alignment and balance performance in professional basketball players. We hypothesized that maintaining a stable occlusion state of the temporomandibular joint (TMJ) through wearing a mouthguard will change the body alignment and improve both static and dynamic balance performance in professional basketball players.

## 2. Materials and Methods

### 2.1. Participants

Twenty-six professional male basketball players who are currently affiliated with the Korean Basketball League voluntarily participated in the study. Participants received an oral explanation of the purpose of the study, the study procedure, the potential risks and benefits, and completed a written consent approved by Institutional Review Board of the university (KHUIRB-019). During the study period, three players were unable to complete the experiment due to personal reasons or orthopedic injury; thus, twenty-three players completed the study. The participants’ average age, career length, height, and body mass were 25.8 ± 8.6 years old, 16.3 ± 5.4 years, 187.5 ± 5.2 cm, and 83.4 ± 8.26 kg, respectively.

### 2.2. Study Procedure

Prior to the experiment, all participants were instructed to visit a designated dentist to undergo an oral examination, temporomandibular disorder test, and diagnostic model test. Subsequently, mouthguards were custom made for players who were determined to have no problem wearing a mouthguard. Participants were familiarized with all the measurements, including body alignment and balance performance tests, at least one week prior to the experiment. A total of three measurements, including body alignment and balance performance tests, were performed with no treatment (without wearing a mouthguard), acute treatments (wearing a mouthguard) and repeated treatments (8 weeks follow-up). There was a seven-day wash-out period between treatments. Participant underwent the measurements without a mouthguard for no treatment and with a mouthguard for acute and repeated treatments. Basketball players were encouraged to wear the mouthguard for at least 3 h a day during strength and conditioning training as well as technical training for the 8-week follow-up period.

### 2.3. Mouthguard

**Figure 1:**
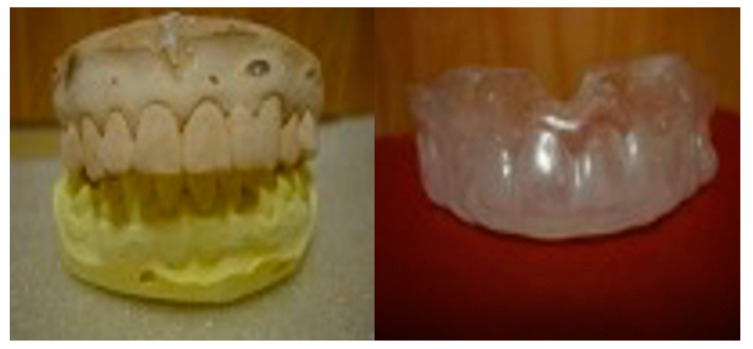
An example of a customized mouthguard.

The customized mouthguard was produced by fabricating a plaster model based on an impression taken with irreversible hydrocolloid impression material on the maxillomandibular dentition of the subjects. Subsequently, Drufomat 2 (Dreve-Dentamid GmbH., Unna., Germany) was used with an ethylene vinyl acetatecopolymer sheet to produce the mouthguard by a traditional laminate method involving thermos compression molding in accordance with the recommendation given by the Korean Academy of Sports Dentistry (Figure 1).

### 2.4. Measurement of Body Alignment

**Figure 2:**
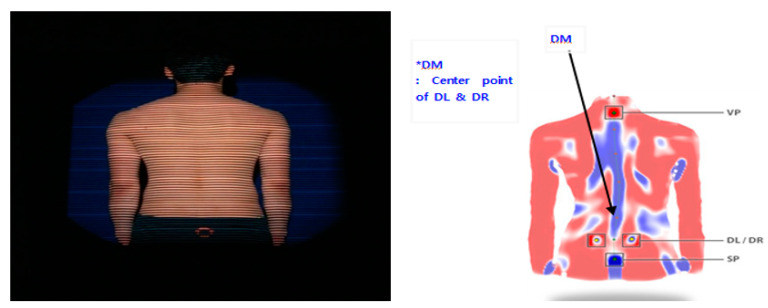
Posture for Formetric III testing and anatomical points (2006).

For spinal and pelvic posture, Formetric Ⅲ3D (Formetric, Diers International GmbH, Schlangenbad, Germany) developed by the Institut fur Experimenteller Biomechanik (University of Münster, Germany) was used, and a videorastereogaphy method was used, where a halogen light source is projected on the back surface of the subject and the image is processed by a raster method. Image acquisition time was approximately 6 s, where the position of four anatomical landmarks—vertebra prominence (VP; C7), sacrom point (SP), left lumbar dimple (DL), and right lumbar dimple (DR)—was automatically established and the values were determined based on anatomical calculations (error: ±0.05 mm). Measurement postures are shown in Figure 2 and the variables used during the measurements are shown in Table 1.

### 2.5. Measurement of Balance Performance

The study measured static balance ability, where the posture was maintained with the body remaining stationary and the center of gravity was maintained on the supporting base without moving. Dynamic balance ability was measured where the posture was maintained while the body was moving. A postural control device (Posturomed 202, Haider Bioswing, Pullenreuth, Germany) was used to measure the left and right postural sway when standing one-legged on the left and right leg. Two measurements, 10 s each, were taken for each posture, with a 1-min rest interval between measurements. The maximum value was recorded.

### 2.6. Statistical Analysis

All data analysis was performed with SPSS for Windows (version 23.0, SPSS Inc., Chicago, IL, USA) and the mean (M) and standard deviation (SD) of all measured values were calculated. A repeated multivariate analysis of variance (MANOVA) analysis with a Bonferroni post hoc test was applied to examine the treatment effects (baseline, acute, and repeated treatment). The adjusted significance level of all statistical values was set at 0.02.

## 3. Results

### 3.1. Changes in Body Alignment

The results of body alignment are presented in Table 2. A total of 10 variables were examined to estimate the spinal and pelvic posture. No significant treatment effect was observed in body alignment (F = 6.476, *p* = 0.302). However, univariate analysis showed a significant difference in pelvic torsion (*p* = 0.001) where both acute (*p* = 0.015) and repeated treatment (*p* = 0.006) of wearing a mouthguard reduced the pelvic torsion compared to no treatment. A significant difference in kyphotic angle was observed between different treatments (*p* < 0.001). The kyphotic angle improved significantly following 8 weeks of wearing mouthguard compared to the baseline (*p* = 0.002) and acute treatment (*p* = 0.001). However, other variables, including trunk torsion, trunk inclination, trunk imbalance, pelvic tilt, pelvic rotation, lordotic angle, surface rotation max, and lateral deviation max, were not different between different treatments.

### 3.2. Changes in Balance Performance

With respect to the measurement results of balance performance, a significant treatment effect was observed across time (F = 3.942, *p* < 0.001). Univariate analysis revealed that almost all categories of static and dynamic balance variables improved significantly after wearing an acute and repeated mouthguard compared to no treatment (*p* < 0.01). Particularly, athletes became more stable following 8 weeks of repeated treatment. The results from analysis of changes in static and dynamic balance are shown in Table 3.

## 4. Discussion

Changes in the body alignment caused by repeated unilateral movements have been recognized as one of the major factors for inducing chronic inflammation, pain, deformation of the vertebrae, and inhibiting the growth of young athletes [20,21]. This study investigated the influence of a customized mouthguard on body alignment and balance performance in professional basketball players. Our findings showed that wearing a customized mouthguard provides a positive effect on balance performance, but no significant effect was observed in body alignment. Notably, repeated treatment over an 8-week period provides a greater impact on balance performance than acute treatment.

Although no significant impact on body alignment was observed in the present study, univariate analyses showed a significant effect on pelvic torsion and kyphotic angle, where it was improved following acute or repeated treatments. Pelvic torsion angle is a factor used to identify pelvic deviation, which refers to the antero-posterior displacement of the left and right pelvis, and as the angle deviates from the normal range of 0°, there is a high probability of abnormal changes. In the present study, the pelvic torsion angle decreased significantly after wearing the mouthguard, which represents the improvement of pelvic balance. It is well understood that the TMJ and nearby muscles share a functional relationship. Abnormalities in TMJ function caused by various factors have been known to weaken the head and neck muscles [22], as well as abdominal and leg muscles. Maintaining safe teeth conditions and jaw repositioning by wearing a customized mouthguard have been shown to improve athletic performance, such as maximal aerobic power, vertical jump, and sprint performance [23].

Kyphotic angle is a variable that reflects the spinal curvature related to the antero-posterior aspect, and is indicative of how much the back is curved towards the front. Relative to the fully curved state, 47°–50° represents the normal range. In the present study, the subjects showed curvature values in the range of 37°–42°, which showed a difference of about 10° as compared to the normal range. Wojtys et al. found an association between the accumulated training time and the increase in thoracic kyphosis and lumbar lordosis [24]. They reported that repeated movements or overload training could cause organic adaptations with potential muscular imbalances and could determine postural changes, which are specific to the practiced modality. These biomechanical compensations may influence the growth processes and lead to the development of various postural patterns [25]. Although the characteristics of each sport may have a major impact on athletes’ postural patterns, excessive exercise load and repetitive motion, such as running and jumping, could cause a limited range of motion and muscle stiffness that could also affect the postural imbalance.

Over the past several years, many studies have been conducted to figure out the determinant factors that can affect the body posture, and the evaluations of respiration and head and neck position have been introduced as determinant factors [26,27]. Recently, studies have reported the role of trigeminal afferences and dental occlusion in proprioception and visual and postural stabilization [28,29]. Sakaguchi et al. [29] examined the association between mandibular position and posture changes, suggesting that occlusal contact may vary during standing and walking if there is a difference in leg length or hip joint or other postural deformations. Another study also reported that body posture could be changed by different dental occlusion positions [30].

Balance or postural stability is a necessary component in both daily activities and sport [31]. Postural stability can be defined as the ability of an individual to remain centered on the ground, which includes feedback from the sensory system that determines continuous neuromuscular changes [32]. Balance ability is associated with the improvement of performance and the reduction of injury factors [33,34]. Contrary to changes in spinal alignment, postural balance ability showed significantly large differences in all categories of static and dynamic balance. Basketball athletes require a high level of balance ability, and the inherent sense of acceptance from the hip is important because of the complexity of the basketball technique. However, various movements, such as deceleration and acceleration, switching direction, and box-out, can develop the unbalanced conditions [35]. Additionally, basketball techniques, including a one-leg jump shoot, foot twist motions, pivot motion, airborne rebounds, and dribbling, include potential risks for developing unbalanced conditions [36]. Therefore, the improvement of balance ability is believed to be of significant help in predicting and maintaining instantaneous movements for basketball players during competitions.

Mouthguards are typical safety devices recommended for use by athletes in various sports to decrease the risk of oral–facial injuries. Basketball players are more likely to hit an opponent in repeated movements such as deceleration, acceleration, jumping, and turning. For this reason, some basketball players use a variety of mouthguards during the season or during training to prevent tooth injuries and protect the maxilla from possible extreme collisions [37]. Recently, as research progresses on the connection between TMJ, the surrounding muscles, and posture, the mouthguard is used as protective equipment, a TMJ occlusion correction treatment device, and equipment to improve performance [14]. Previous studies reported that among various physical fitness factors, wearing a mouthguard can promote functional improvement [38,39]. Previous studies also reported that wearing a mouthguard or mandibular orthopedic repositioning appliance (MORA) had the effect of strengthening head and neck muscles and enhancing leg strength and power performance [40]. Moreover, wearing a mouthguard resulted in a positive change and improvement in the center of posture and balance ability [41], while Yoshinobu et al. reported that just by stabilizing occlusion, balance was maintained quickly, which would allow for an improved sense of balance and postural stability and maintain safe stationary posture after movement [42]. In this study, the repeated treatment of wearing a mouthguard resulted in better static and dynamic balance performance than acute treatment. However, some studies reported that wearing a mouthguard did not improve balance performance in trained men and women [39] and collegiate male athletes [43]. These inconsistent results between the studies may be associated with different subject groups, test equipment, and treatment periods.

Although this study provides substantial information about the implication of mouthguards for body alignment and balance performance among professional basketball players, there are some limitations that need to be considered when interpreting the data. The duration that each athlete wore the mouthguard was not recorded in the present study. Thus, it is difficult to evaluate the dose–response effects. However, basketball players were encouraged to wear the mouthguard by coaches and researchers during strength conditioning and technical training to meet the minimum requirement hours (3 h). This study was a crossover study, but participants were not randomly allocated to the different treatments due to the game schedules, which may have created bias in the results. In future studies, randomized allocation needs to be applied to confirm the effectiveness of mouthguards on body alignment and balance performance in basketball players.

## 5. Conclusions

The present study suggests that wearing a mouthguard improves balance performance. In particular, the repeated treatment of wearing a mouthguard over an 8-week period had a more positive impact on changes in balance performance than acute treatment. Although our findings did not show a significant effect on body alignment, some positive results, such as for pelvic torsion and kyphotic angle, may provide substantial information for developing future longitudinal studies with large sample sizes.

## Figures and Tables

**Table 1:** Body alignment variables.

Variables	Definition	Unit
Torsion Trunk	Left and right rotation of trunk	[°]
Trunk Imbalance	From the VP to vertical line and angle of connect line from VP to DM	[°]
Trunk Inclination	Anterior and posterior inclination of trunk in the side position	[°]
Pelvic Tilt	Length of both pelvic tilts	[mm]
Pelvic Torsion	Contrary anterior and posterior torsion of both pelvic sides	[°]
Pelvic Rotation	Left and right rotation of both pelvic sides	[°]
Kyphotic Angle	Maximum kyphotic angle in the thoracic vertebrae part	[°]
Lordotic Angle	Maximum lordotic angle in the lumbar vertebrae part	[°]
Lateral Deviation	Lateral deviation length to connection line of VP and DM	[mm]
Surface Rotation	Surface rotation angle to connection line of VP and DM	[°]

**Table 2:** Changes in body alignment condition (mean ± SD).

Variables	NT	AT	RT	F-Value	*p*-Value
Trunk torsion [°]	2.0 ± 6.81	1.6 ± 4.11	3.0 ± 4.43	0.782	0.451
Trunk inclination [°]	2.4 ± 2.92	1.8 ± 2.38	1.5 ± 1.83	2.928	0.075
Trunk imbalance [°]	−0.3 ± 1.17	−0.3 ± 0.77	0.04 ± 0.67	2.628	0.098
Pelvic tilt [mm]	−0.7 ± 7.96	0.1 ± 6.62	0.3 ± 4.33	0.423	0.580
Pelvic torsion [°]	4.3 ± 2.93a	2.9 ± 2.38b	2.1 ± 2.01b	9.569	<0.001
Pelvis rotation [°]	−1.8 ± 4.36	−0.5 ± 2.96	−0.3 ± 2.09	3.094	0.063
Kyphotic angle [°]	37.3 ± 6.37a	38.6 ± 6.36a	41.9 ± 5.99b	13.197	<0.001
Lordotic angle [°]	31.7 ± 8.84	33.4 ± 8.56	34.3 ± 9.12	3.223	0.072
Surface rotation max [°]	0.2 ± 7.60	−2.0 ± 5.00	−1.0 ± 4.07	1.979	0.169
Lateral deviation max [mm]	2.4 ± 9.22	1.9 ± 7.52	2.8 ± 6.30	0.314	0.675

Note. Different letters indicate a significant difference between treatments. NT, no treatment; AT, acute treatment; RT, repeated treatment.

**Table 3:** Changes in static and dynamic balance performance (mean ± SD).

Variables		NT	AT	RT	F-Value	*p*-Value
Static	RF X axis	1368.4 ± 812.93a	592.2 ± 477.59b	327.0 ± 211.19c	33.817	<0.001
	RF Y axis	425.1 ± 339.77a	190.3 ± 168.24b	327.0 ± 211.19a	7.665	0.004
	Sum of RF X + Y axis	1502.4 ± 887.03a	660.3 ± 525.93b	374.8 ± 224.90c	33.044	<0.001
	LF X axis	1619.9 ± 1137.99a	504.0 ± 355.42b	258.4 ± 191.59c	31.606	<0.001
	LF Y axis	390.3 ± 305.74a	155.7 ± 94.36b	99.3 ± 52.53c	20.193	<0.001
	Sum of LF X + Y axis	1738.8 ± 1179.47a	563.2 ± 370.38b	300.2 ± 199.55c	33.046	<0.001
Dynamic	RF X axis	1164.6 ± 508.36a	660.0 ± 285.47b	327.0 ± 211.19c	42.657	<0.001
	RF Y axis	446.8 ± 172.96a	363.8 ± 163.83a	272.0 ± 122.69b	16.306	<0.001
	Sum of RF X + Y axis	1400.2 ± 549.12a	847.0 ± 352.71b	595.6 ± 208.65c	40.962	<0.001
	LF X axis	1280.0 ± 680.85a	669.6 ± 363.73b	412.0 ± 214.88c	34.069	<0.001
	LF Y axis	409.5 ± 180.06a	306.0 ± 128.76a	261.8 ± 106.02b	10.670	0.001
	Sum of LF X + Y axis	1449.3 ± 710.18a	819.6 ± 398.35b	562.8 ± 251.46c	32.918	<0.001

Note. RF, right foot; LF, left foot; NT, no treatment; AT, acute treatment; RT, repeated treatment. Different letters indicate a significant difference between treatments.
